# Editorial: Integrated Role of Nutrition and Digestive Physiology for Animal Health

**DOI:** 10.3389/fvets.2021.789496

**Published:** 2021-11-15

**Authors:** Hao-Yu Liu, Johan Dicksved, Anoosh Rakhshandeh, Demin Cai

**Affiliations:** ^1^College of Animal Science and Technology, Yangzhou University, Yangzhou, China; ^2^Department of Animal Nutrition and Management, Swedish University of Agricultural Sciences, Uppsala, Sweden; ^3^Department of Animal and Food Sciences, Texas Tech University, Lubbock, TX, United States

**Keywords:** diet integration, digestive physiology, host-microbe interaction, integrated nutrition, macronutrients

Consisting of nutrition, digestive physiology, and the microbiota, each point of the gut triangle influences the animal metabolic health and performance. The mammalian gastrointestinal (GI) tract is composed of the intestine and multiple accessory organs. To ensure effective digestion, it is organized into a functional structure with specialized and region-specific anatomical, histological, and functional diversities. The morphological and functional features of the digestive system generally reflect the nutritional status of animals. In addition, the abundance of indigenous microbiota plays a pivotal role in the interplays of the triangle of intestine. Given the complexity of the GI tract, we argue that a more holistic perspective should be applied in this research field. Fortunately, with the advent of the next generation sequencing (NGS) technology and other molecular tools, it becomes feasible to gain a better understanding of the bioactive components present in food and feed, and the mechanisms of action toward desired metabolism and overall animal health. This Research Topic “Integrated role of nutrition and digestive physiology for animal health” in Frontiers in Veterinary Science collected 13 scientific contributions from high qualified research groups focusing on large animal species. The articles within this Research Topic detail the gut microbiome signatures in relation to animal metabolism and growth, and cover a wide-range of alternative feed ingredients from their effects on the digestive physiology optimization to the molecular regulation mechanisms, as well as motivations for and use of dietary supplements in livestock industry.

Gopi et al. describe how an early boosting of nutrients can shape the gut physiology and maximize the metabolic potential of broilers. An *in ovo* administration of nucleoside results in a better developed GI function, thus a higher energy metabolizability in chickens when growing up (Gopi et al.). The liver physiology of piglets at birth and at weaning is studied in details by Li et al. where the importance of lipid homeostasis is addressed with an emphasis on epigenetic regulations (Li et al.). In their article, the hepatic cholesterol metabolism of a commercial pig breed Large White is analyzed and compared with a local breed Erhualian, showing differences in several aspects. Biodiversity of livestock populations is necessary for adaptation to climate changes, as well as for consumer demand. However, the metabolic traits of local livestock breeds and their accustomed dietary regimens are often overlooked. Along this line, Meng et al. specifically characterize the beef quality of the Chinese native cattle Yunling, Wenshan, in comparison with Simmental. And provide insights into novel metabolic pathways and the underlying regulatory mechanisms (Meng et al.).

As aforementioned, nutrition is a major key to the efficiency of digestive process and growth performance in animal husbandry. Several different dietary regimens and novel feed resources worldwide are explored. Chen et al. and Shi, Xun et al. critically examine the utilization of dietary antimicrobial alternatives tea tree oil and the combination of curcumin and piperine, their anti-inflammatory properties and the potential to improve animal produce quality (Chen et al.; Shi, Xun et al.). The latter study specifically describes the antioxidant activity of the feed additive and the associated changes of GI function in Wuzhishan piglets (Shi, Xun et al.). This is also underscored in broiler chickens fed onions (*Allium cepa* L.) extract from Egypt and shows less stressful behavior, better GI health and digestibility (Omar et al.). Indeed, oxidative stress can cause overt inflammation and severe damages in animals (Sun et al.). The Egyptian leek (*Allium ampeloprasum var. kurrat*) leaf extract is evaluated as a replacement of antibiotic growth promoter in chickens by Al-khalaifah et al.. Remarkably, both studies in poultry show improved growth performance parameters and economic efficiency including better return and profit. In many developing countries, in-feed antimicrobial usage as a growth promoter is a standard practice in livestock. Repeatedly exposing food animals to small doses of antibiotics contributes significantly to antimicrobial resistance in humans. In this regard, Lourenco et al. investigate the effects of feeding carbadox on the gut microbiome of weanling piglets, showing an antibiotic-dependent shift without growth promoting effects (Lourenco et al.). Nevertheless, researches on antibiotics and the alternatives in livestock science in relation to digestive physiology and metabolic health should be encouraged, especially in countries or areas where antimicrobials can still be purchased over the counter. In addition to feed supplements, non-typical nutrition such as the physi-chemical properties of feed is described by Liermann et al. where interactive effects between dietary rapeseed proportion and technical treatments are shown to alter performance, GI morphometric traits and immune responses in broilers (Liermann et al.).

As the major target of nutrition, and the essential mediator of metabolism, the gut microbiota becomes one area of intense interest. Indeed, livestock health depends on a beneficial host-microbe interaction. Interestingly, Shi et al. reveal a dietary level switch regulation of digestive physiology in black goat kids (Shi, Zhang et al.). Supplementations of coconut oil above the optimal level do not promote growth but suppress rumen microorganisms and their activities, therefore disrupt the intestinal microenvironment. The temporospatial gut microbiota signature is described in angus steers across life span (Welch et al.) and in sheep from the world's highest elevation (Fan et al.), respectively. Apparently, the gut microbiome plasticity supports animals to survive and thrive. In these studies of livestock digestive physiology and microbiota, NGS technologies are widely used. With the advancement of this technology and many other molecular tools readily available for farm animal studies, we are all set to gain a better understanding of the bioactive components present in food and feed, and an integrated perspective of their actions toward desired physiological responses.

## Author Contributions

H-YL wrote the manuscript. JD, AR, and DC edited and contributed to the organization of the editorial articles. All authors contributed to the article and approved the submitted version.

## Funding

This work was supported by the National Natural Science Foundation of China (32002243), Natural Science Foundation of Jiangsu Province (BK20200932), Natural Science Foundation of the Higher Education Institutions of Jiangsu Province (20KJB230001), the Priority Academic Program Development of Jiangsu Higher Education Institutions (PAPD), and Jiangsu Agricultural Science And Technology Innovation Fund [CX(21)2014].

## Conflict of Interest

The authors declare that the research was conducted in the absence of any commercial or financial relationships that could be construed as a potential conflict of interest.

## Publisher's Note

All claims expressed in this article are solely those of the authors and do not necessarily represent those of their affiliated organizations, or those of the publisher, the editors and the reviewers. Any product that may be evaluated in this article, or claim that may be made by its manufacturer, is not guaranteed or endorsed by the publisher.

